# Trust and Perceived Trustworthiness in Health-Related Data Sharing Among UK Adults: Cross-Sectional Survey

**DOI:** 10.2196/83533

**Published:** 2025-11-28

**Authors:** Jonathan R Goodman, Alessia Costa, Richard Milne

**Affiliations:** 1 Society & Ethics, Wellcome Sanger Institute, Wellcome Genome Campus, Hinxton, CB10 1RQ, United Kingdom, 44 01223 834244; 2 Department of Psychiatry, University of Cambridge, Cambridge, England, United Kingdom; 3 Cambridge Public Health, University of Cambridge, Cambridge, England, United Kingdom; 4 RAND Europe, Cambridge, England, United Kingdom; 5 Faculty of Education, University of Cambridge, Cambridge, England, United Kingdom

**Keywords:** trust, trustworthiness, privacy, health data, data sharing

## Abstract

**Background:**

Trust is an essential element in engagement with data sharing and underpins efforts to use data to combat health inequalities. However, research into public trust in data sharing and health care settings may rely on oversimplified notions of what trust entails. How trust relationships manifest in this context has not been widely explored.

**Objective:**

We aimed to establish the primary reasons for the placement of trust and whether these reasons vary by demographics and domain. We also explored the utility of a composite trust score as a predictor for use of technology in the health sphere.

**Methods:**

We conducted a cross-sectional survey using Qualtrics to explore the challenges associated with trust and judgments of trustworthiness in the context of the use of technology to collect health-related data. Participants were recruited using a marketing firm, Dynata, in July 2022 and were UK census matched for population representation. A total of 99.33% (1192/1200) of the target UK-based participants aged ≥18 years (n=605, 50.8% female; n=587, 49.2% male) were asked to rate their level of trust in others generally and in specific entities on an ordinal scale (1-5). We constructed Bayesian cumulative logit models and hierarchical models to evaluate whether demographic characteristics predicted reasons for domain-specific or general trust. We created a composite trust score across health data domains (range 1‐15) and developed models to determine whether this score predicted the likelihood of having used or using a device to track health or well-being. We report all credible intervals at 95%.

**Results:**

General trust responses were bimodally distributed, with the most frequently chosen answers being “usually not” and “usually.” A cumulative logit model suggested that divorced status predicted choosing “almost always not” or “usually not” (β estimate=–0.71, 95% CI –1.17 to –0.28). “They are reliable and keep their promises” and “They behave responsibly” were the most chosen reasons for placing trust. Trust in family, the National Health Service, and technology companies was primarily driven by familiarity, perceived responsibility, and openness and responsible behavior, respectively. A Bayesian hierarchical model suggested that higher general trust was a strong predictor of a higher composite trust score (β estimate=1.93, 95% CI 1.26‐2.59). A higher composite trust score also inversely correlated with the likelihood of having used a device to track health or well-being, whereas higher trust in technology companies and the National Health Service predicted a willingness to use such devices.

**Conclusions:**

Unlike with prior works evaluating trust and trustworthiness, we demonstrate that trust must be understood as context-specific and relational. Policymakers should note that self-reported global trust may not correlate with specific health- and technology-related behaviors and, consequently, that domain-specific measurements of trust are essential in health policy work.

## Introduction

### Background

Trust in the institutions involved in health research and care is consequential, shaping decisions regarding the willingness to make data available for secondary uses, including research [1]. Given the growing interest in the use of digital and data-driven tools in medicine and public health, this has potential wider implications for the ability to deliver effective, equitable services.

In the literature exploring individual-level decisions to share data related to health, researchers tend to place emphasis on the entity being trusted. For example, numerous studies have focused on whether individuals are more likely to share their data with health care practitioners, government bodies, corporations, universities, and so forth (eg, work on trust in scientific and health care institutions is, consequently, broadly concerned with why individuals place trust in specific entities and not others, with emphasis placed on population-level differences in trust placement, such as between countries or between groups within populations, including, eg, between groups based on ethnicity and socioeconomic status [2,3]).

However, to date, the literature on trust in health data has engaged in less detail with what is involved in public trust in data institutions (including the National Health Service [NHS] in England and health technology companies), what members of the public associate with the trustworthiness of these institutions [4,5], and how this relates to the current or future adoption of digital health technologies. This study focused on the qualities or characteristics that are associated with the perceived trustworthiness of the institutions involved in collecting and working with data about health and the relationship between these qualities and those of trustors.

### Institutions That Are Worthy of Trust

A focus on public trust in institutions that collect, use, and share health data treats these institutions implicitly or explicitly as having qualities that are associated (or not) with being worthy of trust. Accordingly, researchers interested in trust have aimed to determine what makes an institution trustworthy; assuming trustworthiness at an institutional level makes sense [6,7]. Qualities associated with trustworthiness in this literature include considerations such as familiarity for both trustor and trustee [8,9], honesty and openness [10], competence [11] and reliability [12], shared values or encapsulated interests [13], good intentions or goodwill [14], and responsibility [15] (“do they act as they should?”).

The aim in this context is not to go into detail on the merits or otherwise of these accounts of trustworthiness but to describe how they highlight a range of qualities, behaviors, and dispositions that are potentially related to trustworthiness, although with little empirical work to unpack their role in everyday life or in how they enable trustworthy institutions and people to distinguish themselves from bad actors [16]. While this movement from philosophical conceptualization into empirical survey presents challenges in terms of the loss of nuance and the openness to interpretation, this literature does offer carefully structured categories that can provide a scaffold for empirical investigation while connecting empirical questions about trust in practice to the normative accounts proposed by Hawley [17]. Following John Dewey’s pragmatist and pluralistic approach [18], the normative concepts of trust can be taken as working hypotheses to be tested, operationalized, and refined in real-world contexts through social research to examine how members of the public interpret and prioritize different grounds for trust. 

Indeed, from the existing literature, it is an open question whether or how philosophical accounts of trust accord with how people place trust in the real world. For example, transparency regarding data use does not appear, in practice, to have the implicit moral content assumed by researchers working in the social sciences and humanities [19]. Instead, it may be linked to negative sentiment insofar as members of the public are not empowered to act on the information they are given about how their data are used. This accords with work by Jones [20] suggesting that desirable qualities, including those that constitute trustworthiness, are not necessarily useful if they are not observable: it is of no benefit to be trustworthy if no one can distinguish one from bad actors (see the work by Goodman and Milne [16] for a discussion on the trustworthiness recognition problem). Being able to recognize the trustworthy is essential to be able to place trust “intelligently” [10].

Moreover, it is not known whether the qualities that people associate with trustworthiness vary with the type of entity seeking trust. While transparency may be a requirement for trust in one entity, such as a government, it does not follow that it is required for trust in other circumstances, such as within a family [21]. This potential inconsistency among desired qualities in trustees suggests that normative discourse regarding trustworthiness, visibility, transparency, and so forth is unlikely to capture important elements of the contextual relationship between trustor and trustee.

The manner in which the relationship between trustor and trustee manifests is also likely to drive behaviors at the individual level. Furthermore, these behaviors may not match academic intuitions about how people categorized into low- or high-trust groups are likely to act, just as increased transparency may not lead to increased trust [22]. Instead, investigating how members of the public self-describe the way in which they trust and under what circumstances and the link of trust relationships between self-reported behaviors is necessary for revealing whether current uses of terms such as “trustworthiness” and “transparency” in the literature predict real-world behavioral patterns.

In line with these insights, we conducted a public survey to explore the challenges associated with trust and judgments of trustworthiness in the context of the use of technology to collect health-related data. This context reflects the growing interest in the use of digital devices to collect health information and the challenges this presents to the distribution of health data between the public and private sectors. Specifically, we aimed to answer the following questions:

1. Which are the primary reasons for the placement of trust, and do these reasons vary by demographics and domain?
2. Do stated general trust—the overall view of whether people can be trusted—and demographic characteristics predict a composite domain-specific trust score?
3. Does a composite trust score predict use of technology in the health sphere?
4. Is trust in technology companies a predictor for using technology for health reasons?
5. Is trust in England’s NHS a predictor for using technology for health reasons?

We hypothesized, in line with previous work, that trust is likely to vary by domain [23] but also by the demographic qualities of the trustor and that the way in which trust is placed is likely to predict whether and how people use data-driven health technologies. If correct, this hypothesis may indicate that trustworthiness manifests and is perceived differently under different conditions and, consequently, should be understood as a function of the context in which the term is used.

## Methods

### Survey Design and Data Collection

We designed the survey on the Qualtrics (Qualtrics International Inc; [24]) platform and used the marketing firm Dynata [25] to recruit a sample of 1200 adults living in the United Kingdom that was representative in terms of educational level, sex, and age between July 20 and 22, 2022. The only inclusion criteria were residence in the United Kingdom to ensure a representative cross-section of the UK population. We developed the measures evaluated for this study only; the measures have not consequently undergone psychometric validation.

The full questionnaire was designed to explore both the likelihood of a person placing trust generally (using a 5-point version of the general trust question [26]) and in a given entity in relation to data about their health (family, the NHS, and health technology companies). The latter 2 of these were chosen to reflect the organizations involved in collecting and using health data, whereas the former represents those proximal to the data subject. Participants were asked to rate their level of trust in others generally (“Generally speaking, would you say that people can be trusted?”) and in specific entities (“Do you feel that you can generally trust the NHS with data about your health?”) on an ordinal scale (1-5) that included “almost always not,” “usually not,” “can’t choose,” “usually,” and “almost always.”

We also explored the reasons why people place trust generally or specifically in relation to health data in their family, the NHS, or health technology companies, which could be any or all of the following: (1) “They do what they do with the best of intentions,” (2) “They are good at what they do,” (3) “They behave responsibly,” (4) “They are reliable and keep their promises,” (5) “They would not try to take advantage of me if they could,” (6) “They are open about what they are doing,” (7) “They share my values,” and (8) “They are familiar to me.”

These questions draw on previously published research aiming to explain the placement of trust [27], informed and adapted to reflect the diverse conceptual literature on trustworthiness. In the widely adopted ABI model of trust [28], for example, trustworthiness has often been associated with ability (being good at what they do), benevolence (having the best of intentions), and integrity (behaving responsibly and not taking advantage). As discussed previously, the wider sociological and philosophical literature highlights considerations such as familiarity, honesty and openness, competence and reliability, shared values or encapsulated interests, good intentions or goodwill, and responsibility.

We also asked whether participants were likely to use or own technological tools that could theoretically be used to analyze personal health data or provide tailored information about one’s health. Finally, we collected demographic data, which included age, gender, educational level, caring status (whether the participant self-identifies as a carer), relationship status, and whether the participants had children.

### Ethical Considerations

This study received human participant research ethics approval through the research ethics process of the Faculty of Education Ethics Committee at the University of Cambridge in July 2022. We received written informed consent from all participants at the start of the survey; recruitment was conducted between July 20 and 22, 2022. Participants received a small cash compensation (less than £1 [US $1.31]) through Dynata for their time. All study information was anonymous as no personally identifying information was collected during the survey, including IP addresses. No identification of individual participants in any images of the manuscript or in the supplementary material is possible. All data were collected and stored using the University of Cambridge’s Qualtrics survey platform, which is General Data Protection Regulation compliant and International Organization for Standardization 27001 certified. More details can be found in Qualtrics [29].

### Analysis

We evaluated the demographic data and general responses to the online questionnaire using base functions in R using RStudio (Posit PBC) [30] and created visualizations of the data using the *ggplot2* package for R (R Foundation for Statistical Computing) [31]. We then created linear models in a Bayesian framework using the *brms* package [32] relying on Markov chain Monte Carlo sampling. In our first set of models, we evaluated whether participant demographic characteristics were predictive of the likelihood of higher general trust or placing trust in family, the NHS, or health technology companies. We visualized the predictions of these models using the *sjPlot* package in R [33]. Missing data occurred in less than 1% of cases across the dataset.

Next, we evaluated whether the reasons for the placement of trust differed by domain and for general trust. To do this, we plotted the most frequently given reasons for why the participant would place trust in family, the NHS, and health technology companies and in general. We constructed Bayesian hierarchical models (an alternative, probability-based method for analysis akin to mixed-level frequentist models) using participants as a random effect to evaluate whether demographic characteristics predicted reasons for domain-specific or general trust. Full information about the analysis method, including code for replication, is available open source.

Finally, we created a composite trust score across health data domains (ie, excluding general trust) where participants could score between 1 and 15. A score of 1 indicates low trust across the domains of family, the NHS, and health technology companies, whereas a score of 15 indicates very high trust across these domains. We developed our final set of models to determine whether a composite trust score predicted the likelihood of having used or using a device to track health or well-being [34].

## Results

### Demographics

Overall, 99.33% (1192/1200) of the target participants took part in the survey; full demographic data from our sample are available in Table 1. All participants were aged at least 18 years, with both age ranges and sexes balanced in the sample. Nearly all participants (1186/1192, 99.49%) had completed at least secondary schooling, whereas 60.4% (720/1192) were married, in a civil partnership, or living with a partner. A small subset of participants (174/1192, 14.59%) self-described as carers.

**Table 1.** Demographic characteristics of adults aged ≥18 years recruited via Dynata for a UK-representative survey conducted on July 20 to 22, 2022, exploring trust in health data sharing across family, the National Health Service, and health technology companies (N=1192).

Characteristic	Participants, n (%)
Age (years)	
18-24	145 (12.21)
25-34	233 (19.53)
35-44	239 (20.19)
45-54	225 (18.91)
55-64	196 (16.41)
≥65	153 (12.83)
Unknown	1 (0.15)
Sex	
Male	587 (49.21)
Female	605 (50.89)
Educational level	
Primary school	6 (0.5)
Secondary school	554 (46.51)
University degree or equivalent	487 (40.96)
Postgraduate degree	145 (12.24)
Relationship status	
Married, civil partnership, living with a partner	720 (60.44)
Widowed	32 (2.71)
Divorced	75 (6.32)
Separated	17 (1.41)
Single	348 (29.11)
Children	
Yes	672 (56.43)
No	517 (43.42)
Self-described as carers	
Yes	174 (14.61)
No	1015 (85.24)

### General Trust

When asked generally whether people can be trusted (“Generally speaking, would you say that people can be trusted?”), the responses were bimodally distributed, with the most frequently chosen answers being “usually not” and “usually” (each encompassing >400 of the 1192 total responses; ), in accordance with previous research [35].

Initial analysis using a Bayesian cumulative logit model suggested that age, gender, and educational level did not predict general trust; however, divorced status predicted choosing “almost always not” or “usually not” (β estimate=–0.71, 95% CI –1.17 to –0.28; ).

When asked to indicate the 3 most important reasons for placing trust in others (“In general, when you think about whether you can trust someone, which of these reasons would you say were the three most important?”), the most chosen response was “They are reliable and keep their promises” (771/1192, 64.7% of cases), followed by “They behave responsibly” (629/1192, 52.8% of cases). “They share my values” was chosen the least (264/1192, 22.1% of cases).

A Bayesian hierarchical model using participant as a random effect suggested that most demographic characteristics were not predictive of reasons for the placement of trust, although older age groups were linked to a greater likelihood of choosing “They are familiar to me,” which was otherwise chosen less frequently among the cohort.

### Trust in Family

Most participants (951/1192, 79.78%) indicated that they would “usually” or “almost always” trust family with their health data (Table 2). Fewer than 50 participants each, in contrast, chose “almost always not” (17/1192, 1.43%) or “usually not” (46/1192, 3.86%). A Bayesian cumulative logit model suggested that only single status predicted a lower trust rating for family (β estimate=−0.39, 95% CI −0.66 to −0.13).

**Table 2.** Distribution of responses for general trust and trust in family, the National Health Service (NHS), and health technology companies among 1192 UK adults surveyed on July 20 to 22, 2022, using a 5-point ordinal scale.

Response option	General, n (%)	Family, n (%)	NHS, n (%)	Technology companies, n (%)
“You almost always can’t be too careful in dealing with people”	208 (17.45)	17 (1.43)	31 (2.6)	97 (8.15)
“You usually can’t be too careful in dealing with people”	423 (36.07)	46 (3.87)	77 (6.47)	187 (15.63)
“Can’t choose”	87 (7.3)	178 (14.9)	172 (14.44)	376 (31.51)
“People can usually be trusted”	429 (35.99)	439 (36.87)	571 (47.86)	398 (33.36)
“People can almost always be trusted”	38 (3.19)	512 (42.93)	341 (28.63)	136 (11.34)

Regarding the reasons primarily chosen for placing trust in family regarding health data (“Thinking about whether your family can be trusted with data about your health, which three of these reasons for trusting would be most important to you?”), participants most frequently chose familiarity (“They are familiar to me”) and least frequently chose “They are good at what they do.” A Bayesian hierarchical model suggested that older participants (aged 55-64 and ≥65 years) were least likely to select “They are good at what they do,” although the estimates were not strong.

### Trust in the NHS

As with family, most participants selected “usually” or “almost always” when asked, “Can the NHS be trusted with your health data?” (Table 2). Compared with having a primary school education, having a secondary school education (β estimate=1.96, 95% CI 0.64‐3.29), university degree or equivalent (β estimate=1.83, 95% CI 0.51‐3.15), and postgraduate degree (β estimate=2.08, 95% CI 0.71‐3.43) predicted increased trust in the NHS, with similar relative estimates among those groups. “They behave responsibly” was the primary reason chosen for trust in the NHS, whereas “They share my values” was chosen the least frequently. Older age groups were less likely to choose “They share my values”.

### Trust in Health Technology Companies

Compared with family and the NHS, fewer participants chose “almost always” when asked, “Can tech companies be trusted with your health data?” (Table 2); most participants chose “usually,” followed by “can’t choose.” Single relationship status was a weak predictor for lower trust in these entities (β estimate=−0.35, 95% CI −0.61 to −0.10). “They behave responsibly” and “They are open about what they do” were the most frequently chosen reasons for trust in this setting. “They share my values” was the least frequently chosen. A Bayesian hierarchical model evaluating reasons did not suggest any strong relationships between demographic variables and the reasons for trust in health technology companies.

### Composite Score

We next created a composite score (1-15; minimum trust=1; maximum trust=15) adding together self-scores on the domain-specific questions on trust (family, the NHS, and health technology companies; 1-5 each). A Bayesian cumulative logit model suggested that higher general trust was a strong predictor of a higher composite trust score (β estimate for a self-score of 5 on general trust vs 1=1.93, 95% CI 1.26‐2.59). Single relationship status (β estimate=−0.31, 95% CI −0.56 to −0.07) was the only demographic variable that predicted the composite trust score and was both weak and negative.

Categorizing participants by composite score as low trustors (score of 1-5; 65/1192, 5.5% of the participants), medium trustors (score of 6-10; 755/1192, 63.3% of the participants), or high trustors (score of 11-15; 365/1192, 30.6% of the participants) suggested that, across domains, reliability was a highly rated reason for the placement of trust, although low trustors placed slightly more emphasis on responsibility. Shared values and familiarity, in contrast, were consistently chosen as the least important reasons across the 3 groups.

**Figure 1.**
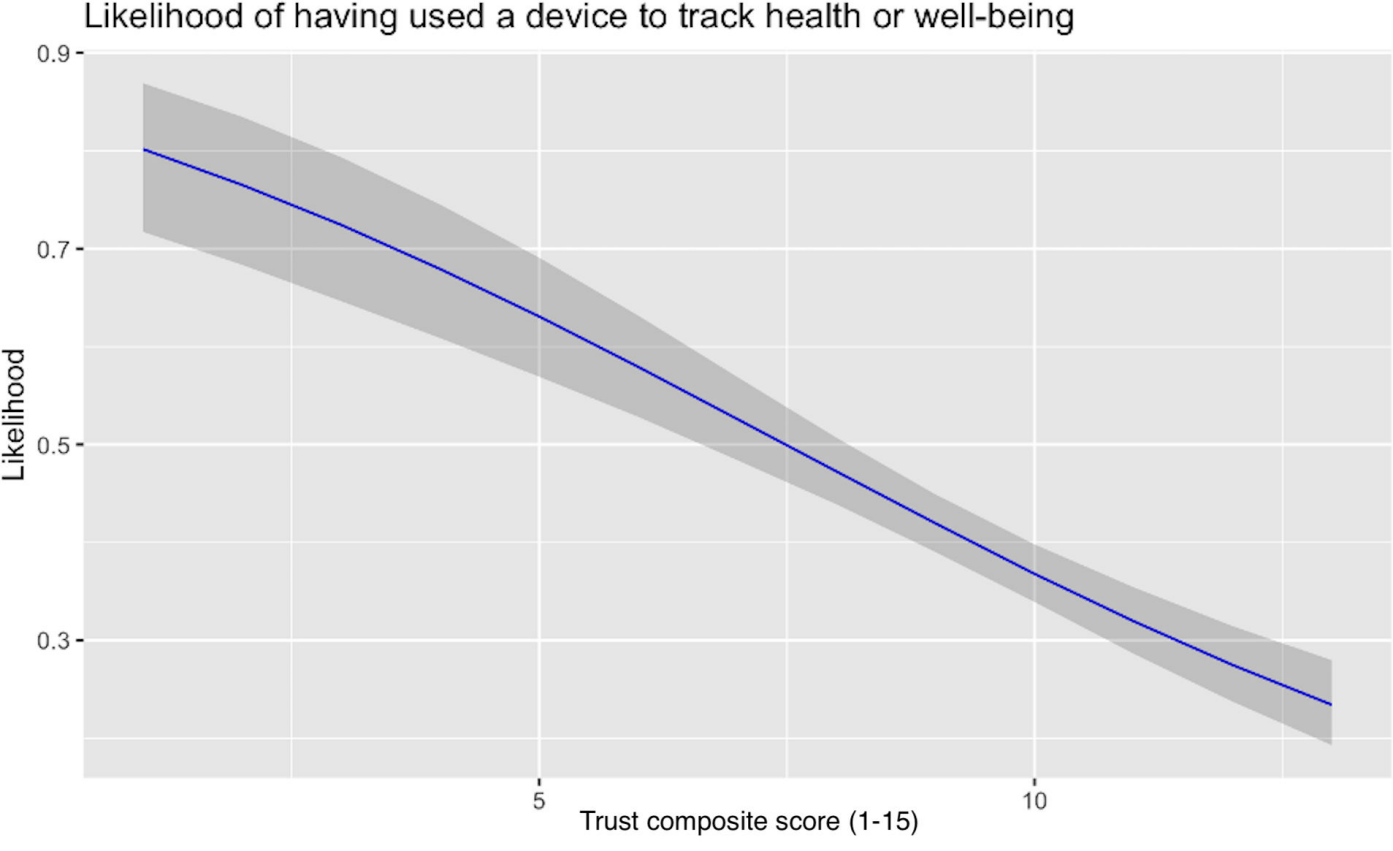
Relationship between composite trust score and previous use of health technology tools among 1192 UK adults surveyed on July 20 to 22, 2022. A higher composite trust score inversely correlated with the likelihood of having previously used a device to track health or well-being.

Figures1^3^ show the relationships, respectively, between composite trust and previous use of health technology tools, trust in technology companies and the likelihood of using a health technology tool, and trust in the NHS and the likelihood of using a health technology tool. The first of these relationships was strong and negative; the other 2 were strong and positive. We discuss the compatibility of these findings in the following section.

**Figure 2.**
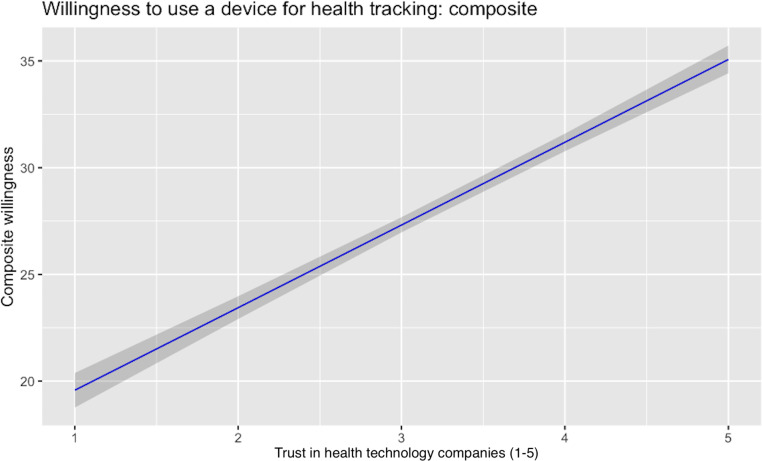
Relationship between trust in health technology companies and willingness to use health technology tools among 1192 UK adults surveyed on July 20 to 22, 2022. A higher trust in technology companies predicted a greater willingness to use a device for health tracking.

**Figure 3.**
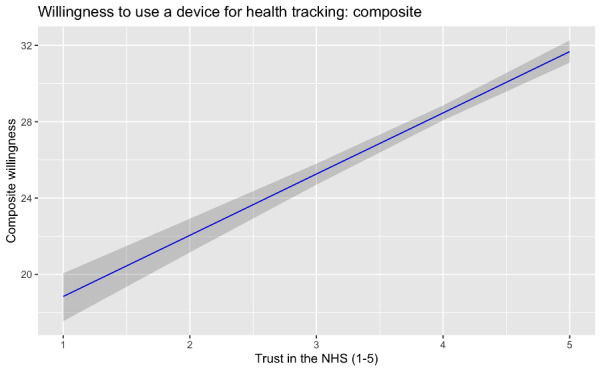
Relationship between trust in the National Health Service (NHS) and willingness to use health technology tools among 1192 UK adults surveyed on July 20 to 22, 2022. A higher trust in the NHS predicted a greater willingness to use a device for health tracking.

## Discussion

### Principal Findings

In this study, we aimed to determine how qualities associated with trustworthiness differ depending on the relationship between trustor and trustee in the context of health data use. Unlike with prior works evaluating trust and trustworthiness that treat trust at the global rather than contextual level, we demonstrated that trust must be understood as both context-specific and relational. Efforts aiming to build public trust in health governance and beyond should account for this relational quality in academic discourse, policy, and communication.

For example, while several reasons for trust, such as perceived reliability, may be rated by members of the public as important with regard to health data, in specific domains, such as whether family should be trusted with one’s health data, familiarity may be the primary reason for trust placement. Moreover, while general trust appeared to correlate with a composite trust score, a high composite trust score did not predict device use. Instead, domain-specific trust measurements such as trust in technology companies predicted device use.

The situated and contextual nature of trust relationships suggested by this finding accords with qualitative and ethnographic work that shows that trust emerges contextually by domain and depending on the relationship between trustor and trustee [36,37]. This presents a challenge to efforts to establish accounts of trust that are not only normative but also functionally relevant. The question of how best to place trust, then, is more complex than assuming that individuals will trust others who demonstrate trustworthiness generally: the manner in which trustworthiness must be demonstrated is a function of the trust relationship and the broader information environment.

Within a domain such as health data sharing, for example, about which there is a large body of published literature and media reports (see the work by Baines et al [38] for a review), how individuals decide whether a given institution is trustworthy will depend not only on factors such as the institution’s overall reputation but also on the qualities that a specific trustor believes this type of trustee should have.

Accordingly, while members of the public may have specific concerns about the potential problems that could emerge from organizations having access to health data [5], the placement of trust may be driven not entirely by these concerns but rather by the trustor’s qualities in relation to the trustee. Trustworthiness with health data, in this regard, is an underlying quality [16] that manifests and is perceived differently under different conditions: trusting one’s family may be a question of familiarity, trusting the NHS may be a question of perceived responsibility, and trusting corporations may be a question of perceived transparency.

However, when participants were grouped into clusters of low, medium, and high trust in relation to the 3 types of trustees, there were consistent qualities that were rated highly or lowly as reasons for placing trust [7]. Thus, responsibility and reliability persisted as primary reasons for trust across groups, whereas shared values and familiarity were less important—despite familiarity being the primary reason chosen for trust in family members. Therefore, valuating the reasons for trust in the absence of understanding the relationship between trustor and trustee may obfuscate important elements of why trust is placed.

Interestingly, the consequences of trust suggested by these findings are complicated. Thus, whether an individual self-rates as trusting across the family, NHS, and health technology company domains may be predictive of intended behaviors, such as previous use of health technology tools or apps, but not of existing behaviors. One possibility is that this reflects the different social and political positions associated with digital health. Over the last 15 years, the widespread adoption of digital health tools has been closely associated with the move toward self-tracking and the “quantified self” movement. This associates digital health devices with individual empowerment, responsibility, liberation from corporate or system control [39], and “soft resistance” to data aggregation [40]. Therefore, adoption of health technologies may be perceived to be in tension with institutionalized and solidaristic forms of health care [41]. In some cases, for example, the extensive adoption of digital self-monitoring is celebrated for its ability to permit forms of self-care that cannot be provided by health systems or that enable individual control of data about health [39]. As a result, we might expect those individuals who conform most closely to the model of those who “generate data about themselves for themselves” [42] to lie at an extreme of high adoption and low trust in institutions involved in health care. Conversely, those who have the most trust in the wider health system may feel a greater willingness to rely on it and lower need to monitor their own health.

In contrast, the willingness of “high trustors” to use such tools in the future if proposed by the NHS reflects a differing logic of adoption. In this case, digital tools are part of rather than in addition or opposition to health systems. This disjuncture between existing and future adoption poses interesting questions and challenges for solidarity-based efforts to ground the introduction of digital and data-driven technologies into health systems [43].

However, it is important to note that there are other barriers to the use of these tools that do not involve trust. These barriers may include the potential benefits these tools might offer or the lack thereof. If, for example, an individual is willing to entrust personal health data to a company but does not believe that the benefits of using the tool are sufficient to purchase one, trust is irrelevant to use.

The findings suggest a need for a conceptual account of trust that accounts for domain-specific relationships such as those in the context of health data and a series of questions that are pertinent to determining whether trust is likely to exist between 2 parties. These may include the following: (1) what is the trustor’s relationship to the potential trustee? (2) Is the trustor in a position to exploit the trustee without severe repercussions? (3) What benefits may the trustor gain from sharing information with the trustee?

Answering these questions in a given relationship will help determine whether trust is or ought to be placed and further highlight, following the results of this study, the reasons likely to be important to the trustor in making a decision about the trustee. Policymakers should note that self-reported global trust may not correlate with specific health- and technology-related behaviors and, consequently, that domain-specific measurements of trust are essential in health policy work.

### Limitations

This study has several limitations common to online survey work. First, owing to the fact that trust is a sensitive topic with moral content, participants may have misrepresented their own views, leading to a self-report bias. Second, owing to the fact that this study was conducted online, it was impossible to obtain a fully representative participant sample, including, for example, individuals who do not have access to a computer or smartphone. Finally, the survey’s quantitative structure made it impossible to capture individual views underlying their choices. Each limitation suggests the need for further, more in-depth studies on this topic, which we are currently planning.

### Conclusions

We suggest—in contrast with works to date on trust in the health care context that broadly consider trust at a global level only—the need for a broader project working toward a comprehensive framework of trust that captures its contextual and relational nature. This would enable a better understanding of how trust is placed in practice in the context of health data and more broadly. For more effective data governance, understanding that a broad metric of trust is unlikely to capture the intricacies of the trustor-trustee relationship is a critical insight that must be explored further. We believe that global accounts of individual-level trust can be improved at a policy level by developing a measurement of trust that more closely mirrors the contextual manifestation of trust relationships outlined in this paper. In future work, we aim to outline a specific framework for doing so effectively. Finally, more data are necessary to uncover the varying reasons people have for placing trust in varying kinds of trustees, accounting for complex interplays such as whether trustees are individuals or institutions.

## Abbreviations

NHS: National Health Service
